# High-Performance AlGaN Double Channel HEMTs with Improved Drain Current Density and High Breakdown Voltage

**DOI:** 10.1186/s11671-020-03345-6

**Published:** 2020-05-20

**Authors:** Yachao Zhang, Yifan Li, Jia Wang, Yiming Shen, Lin Du, Yao Li, Zhizhe Wang, Shengrui Xu, Jincheng Zhang, Yue Hao

**Affiliations:** 1 State Key Discipline Laboratory of Wide Band Gap Semiconductor Technology, School of Microelectronics, Xidian University, No.2 South TaiBai Road, Xi’an, 710071 China; 2 Shanghai Academy of Spaceflight Technology, Shanghai, 201109 China; 3 Shanghai Precision Metrology and Testing Research Institute, Shanghai, 201109 China; 4 Department of Electronic Engineering, Xi’an University of Technology, Xi’an, 710048 China; 5 China Electronic Product Reliability and Environmental Testing Research Institute, No. 110 Dongguanzhuang Road, Guangzhou, 510610 China

**Keywords:** Nitride, High electron mobility transistor, Double channel, AlGaN channel, Breakdown

## Abstract

In this work, AlGaN double channel heterostructure is proposed and grown by metal organic chemical vapor deposition (MOCVD), and high-performance AlGaN double channel high electron mobility transistors (HEMTs) are fabricated and investigated. The implementation of double channel feature effectively improves the transport properties of AlGaN channel heterostructures. On one hand, the total two dimensional electron gas (2DEG) density is promoted due to the double potential wells along the vertical direction and the enhanced carrier confinement. On the other hand, the average 2DEG density in each channel is reduced, and the mobility is elevated resulted from the suppression of carrier-carrier scattering effect. As a result, the maximum drain current density (*I*_max_) of AlGaN double channel HEMTs reaches 473 mA/mm with gate voltage of 0 V. Moreover, the superior breakdown performance of the AlGaN double channel HEMTs is also demonstrated. These results not only show the great application potential of AlGaN double channel HEMTs in microwave power electronics but also develop a new thinking for the studies of group III nitride-based electronic devices.

## Introduction

Group III nitride-based high electron mobility transistors (HEMTs) have been identified as the most promising candidate for next-generation microwave power electronics owing to their fast-switching speed and low-switching loss [1–5]. Lately, the most advanced nitride HEMTs have achieved initial commercialization up to 650 V. However, with the maturity of device fabrication technology, it has become increasingly difficult to further scaling up the breakdown voltages (*V*_b_) and improving the device reliability at high temperatures. Therefore, in view of the larger bandgap and superior thermal stability of AlGaN over GaN, AlGaN channel devices have been proposed as promising candidate to further improve the performance limits of nitride HEMTs in high-voltage and high-temperature applications [6–15].

Nanjo et al. demonstrated the remarkable breakdown voltage enhancement of AlGaN channel HEMTs, and the obtained maximum breakdown voltages were 1650 V in the Al_0.53_Ga_0.47_N/Al_0.38_Ga_0.62_N HEMTs with the gate-drain distances of 10 μm [6]. Afterwards, Nanjo et al. further promoted the breakdown voltage of the Al_0.40_Ga_0.60_N/Al_0.15_Ga_0.85_N HEMTs to 1700 V [8]. Zhang et al. fabricated the AlGaN channel HEMTs with a novel ohmic/Schottky-hybrid drain contact, and a record high breakdown voltage of more than 2200 V was obtained for the AlGaN channel HEMTs [11]. Xiao et al. proposed the AlGaN channel heterostructures with high 2DEG mobility of 807 cm^2^/V·s, and the records of maximum drain current and I_on_/I_off_ ratio were reported for the fabricated AlGaN channel HEMTs [14]. Whereafter, Xiao et al. proposed the normally off HEMTs with superlattice AlGaN channel layer for the first time, and the fabricated devices showed a breakdown voltage over 2000 V, a high on current density of 768 mA/mm, and a threshold voltage (*V*_T_) of 1.0 V [15]. Recently, Baca et al. evaluated the radio frequency (RF) performance of AlGaN channel HEMTs with 80-nm long gate. The f_T_ of 28.4 GHz and f_MAX_ of 18.5 GHz were determined from small signal S-parameter measurements [12]. These results illustrate the promise of AlGaN channel HEMTs for RF power applications.

However, the limitations of the previously reported AlGaN channel devices are equally obvious. On one hand, on account of the ternary alloy disordered scattering effect, the two dimensional electron gas (2DEG) mobility in AlGaN channel is much lower than that in GaN channel. As a result, the current drive capacity of AlGaN channel devices is much lower than that of the traditional GaN channel devices. On the other hand, in order to induce the same amount of 2DEG in AlGaN channel, the AlN component in AlGaN barrier layer should be higher than that of conventional GaN channel heterostructures, which will increase the difficulties in material growth process. These contradictions seriously inhibit the widespread application of AlGaN channel devices, and the optimizations of heterostructure layout are urgently needed.

Double channel technique is an intriguing approach to promote the channel carrier density of nitride-based heterostructures without any adverse impact on the electron mobility, and the current conduction capability of the devices will be improved [16–18]. However, there have been few reports on the AlGaN double channel heterostructures or electron devices up to now. In this work, for the first time, AlGaN double channel heterostructure is proposed and grown to resolve the contradictions between the current drive capability and the breakdown performance of nitride-based electron device. Further, high performance AlGaN double channel HEMTs based on the novel heterostructure are fabricated and investigated in detail.

## Methods

**Fig. 1:**
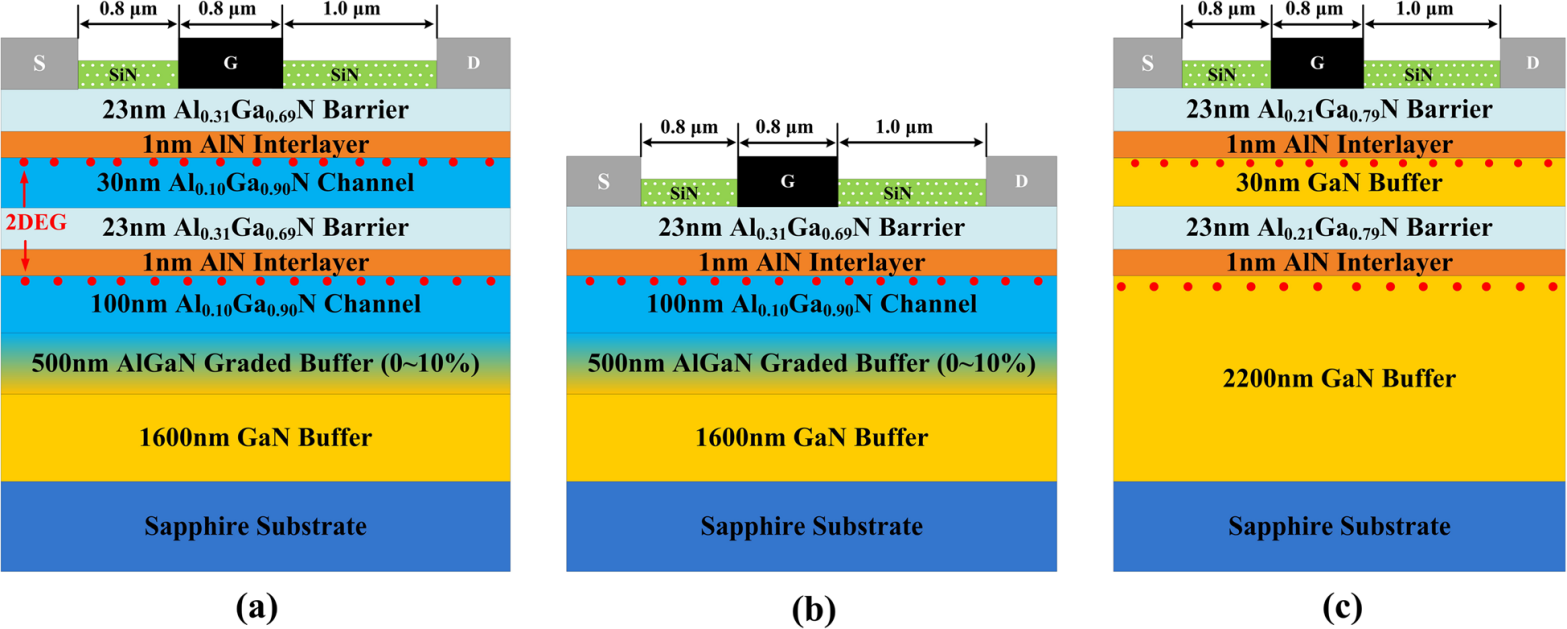
Cross-sectional view (not to scale) of **a** AlGaN double channel, **b** AlGaN single channel, and **c** GaN double channel heterostructures (HEMTs)

**Fig. 2:**
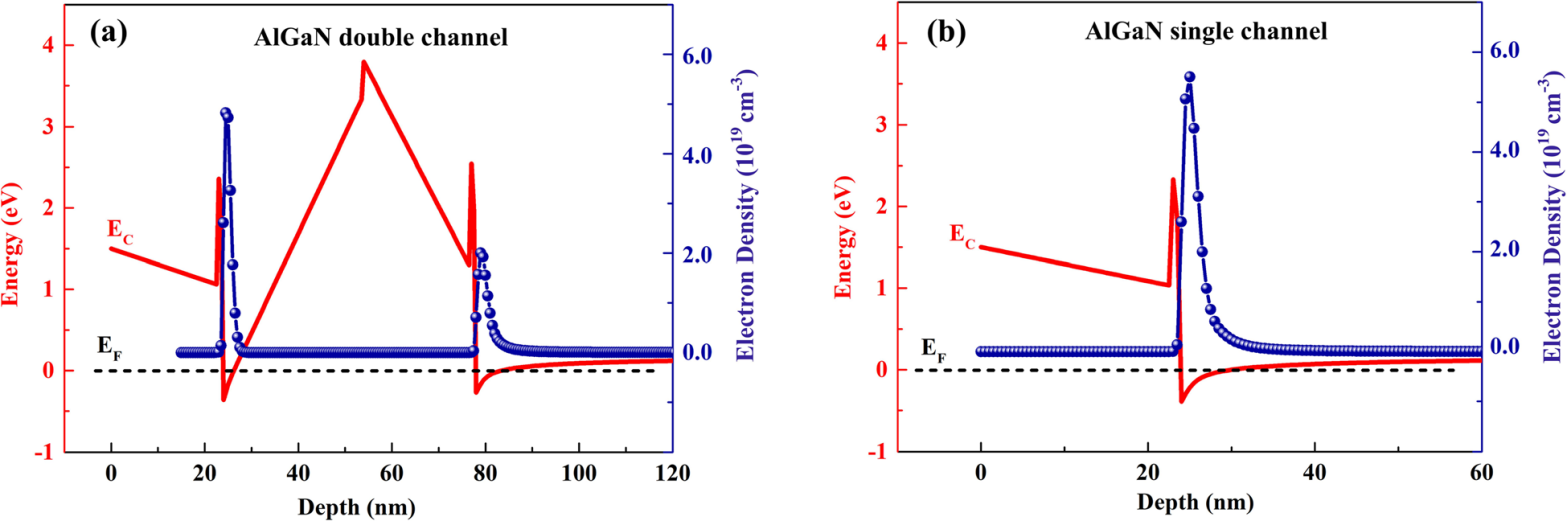
Conduction band diagrams and electron density distributions of AlGaN double channel and single channel heterostructures

The cross-section schematic of the AlGaN double channel heterostructure is shown in Fig. 1a, and the growth processes can be summarized as follow. Firstly, 1600 nm GaN buffer layer was grown on the sapphire substrate. Then, 500 nm graded AlGaN buffer layer with AlN composition increasing from 0 to 10% was grown, which was beneficial to suppress the formation of parasitic channel. Whereafter, 100 nm lower AlGaN channel, 1 nm AlN interlayer, and 23 nm lower AlGaN barrier were grown successively, and the AlN compositions in the channel and barrier layers are 10% and 31%, respectively. Finally, 30 nm upper AlGaN channel, 1 nm AlN interlayer, and 23 nm upper AlGaN barrier layers were grown, for which the compositions were the same with the lower layers. The conduction band diagram of the AlGaN double channel heterostructure can be calculated by self-consistently solving the one dimensional Poisson-Schrödinger equation, which employs the finite-difference method with a nonuniform mesh size [19]. The conduction band diagram and the extracted electron density distribution of the AlGaN double channel heterostructure are illustrated in Fig. 2a, and the results of AlGaN single channel heterostructure are also provided in Fig. 2b for composition. Two deep potential wells are formed at the interface of AlN interlayers and Al_0.10_Ga_0.90_N channel layers for the AlGaN double channel heterostructure, corresponding to the double 2DEG channels. In addition, it can be observed that the 2DEG density in upper channel is higher than that in lower channel, which can be explained from two aspects. On one hand, the lower AlGaN barrier acts as back barrier of the upper channel, which is beneficial to promote the 2DEG confinement of upper channel. On the other hand, the main supplying source of the channel 2DEG in nitride heterostructures is the donor-like surface states [20], which are more close to the upper channel.

## Results and Discussion

**Fig. 3:**
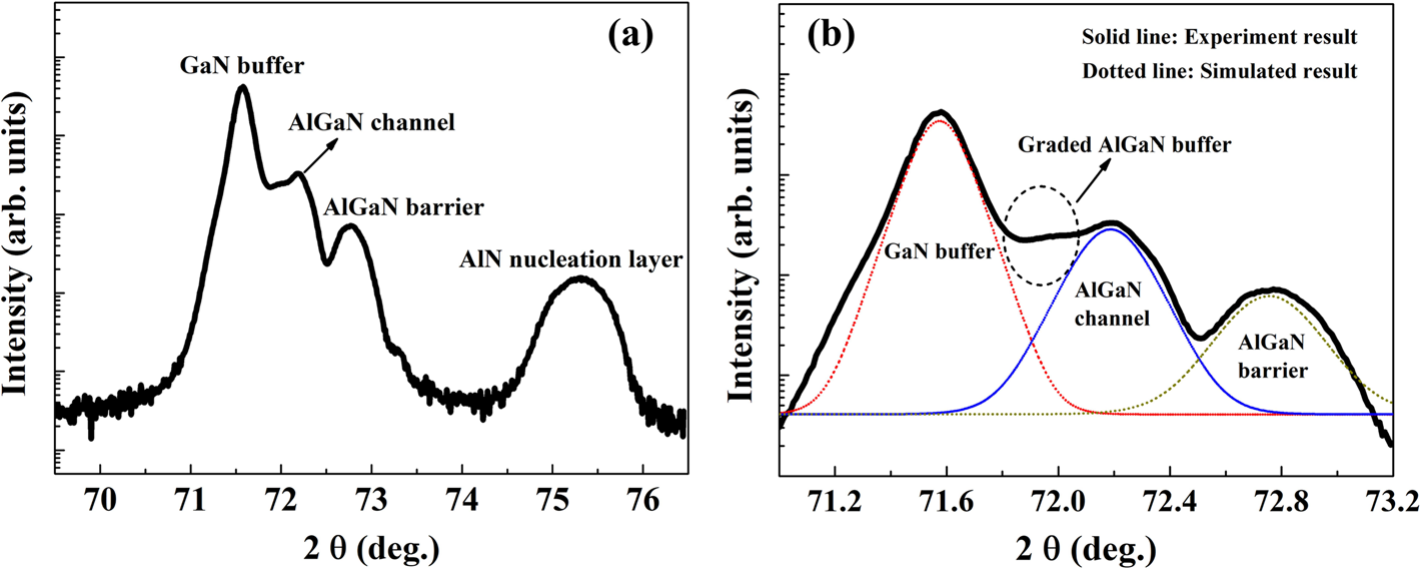
HRXRD (0004) plane ω-2θ scan of AlGaN double channel heterostructure

Figure 3 displays the high resolution x-ray diffraction (HRXRD) ω-2θ scan result of the AlGaN double channel heterostructure from symmetric (0004) reflection. The diffraction intensity from AlN nucleation layer, GaN buffer, AlGaN graded buffer, AlGaN channel, and AlGaN barrier layers can be observed. Moreover, the spectrum scan from 71.0 to 73.2° is presented in Fig. 2b with a magnification for clarity, and Lorentz function is applied to fit the multi-peaks. The diffraction peaks of GaN buffer, AlGaN channel, and AlGaN barrier locate at 71.6°, 72.2°, and 72.8°, and the AlGaN graded buffer results in a platform between the peaks of GaN buffer and AlGaN channel. These results indicate the distinct multi-layer structure and the sophisticated control of the growth process, and the AlN compositions of 10% and 31% in the AlGaN channel and barrier can be extracted.

**Fig. 4:**
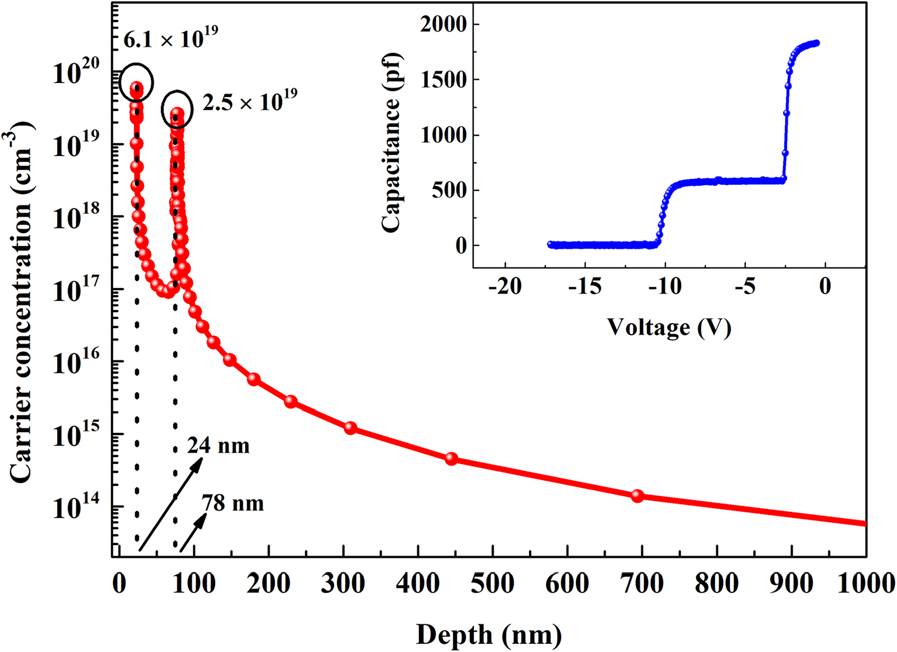
C-V characteristics and electron distribution curve of AlGaN double channel heterostructure

Capacitance-voltage (C-V) measurement with mercury-probe configuration was performed to investigate the double channel characteristics of the heterostructure. As shown in the inset of Fig. 4, two distinct capacitance steps can be observed at around − 2.5 V and − 10 V with the applied voltage swept from 0 to − 15 V, corresponding to the depletion of 2DEG at AlN/Al_0.10_Ga_0.90_ interfaces. In addition, the carrier distribution properties can be extracted from C-V curve and the result is illustrated in Fig. 4. Two carrier concentration peaks locate at 24 and 78 nm with the values of 6.1 × 10^19^ and 2.5 × 10^19^ cm^−3^, which is in accordance with the calculated result as shown in Fig. 2. Specially, no parasitic conduction channel can be observed until the test depth reaches 1 μm, suggesting the successful achievement of double channel properties of the heterostructure. In addition, the 2DEG sheet density and mobility were determined to be 1.3 × 10^13^ cm^−2^ and 1130 cm^2^/V∙s by the Hall effect measurement.

The standard HEMTs fabrication process was carried on the AlGaN double channel heterostructure. The device fabrication process started with ohmic contact formed with Ti/Al/Ni/Au multilayer metal stack deposited by electron beam evaporation, followed by a rapid thermal anneal at 850 °C for 30 s in N_2_ atmosphere. Then, the mesa isolation was performed by Cl_2_/BCl_3_ inductively coupled plasma etching to a depth of 200 nm, and 100-nm-thick SiN passivation layer was formed by plasma-enhanced chemical vapor deposition. Afterwards, a gate area with a length (*L*_G_) of 0.8 μm was defined by photolithography after etching the top SiN with CF_4_ plasma, and then a Ni/Au schottky gate electrode was deposited. The gate-source (*L*_GS_) and gate-drain (*L*_GD_) distances are 0.8 and 1 μm, respectively. For comparison purposes, two additional HEMTs samples based on the conventional AlGaN single channel and GaN double channel heterostructures were also fabricated, and the cross-section schematics are shown in Fig. 1 b and c. The device process and characteristic parameters of the additional HEMTs samples are exactly the same with the AlGaN double channel HEMTs. The output and transfer properties of the devices were carried out with Keithley 4200 semiconductor parameter analyzer, and the breakdown characteristics were performed using Agilent B1505A high-voltage semiconductor analyzer system.

**Fig. 5:**
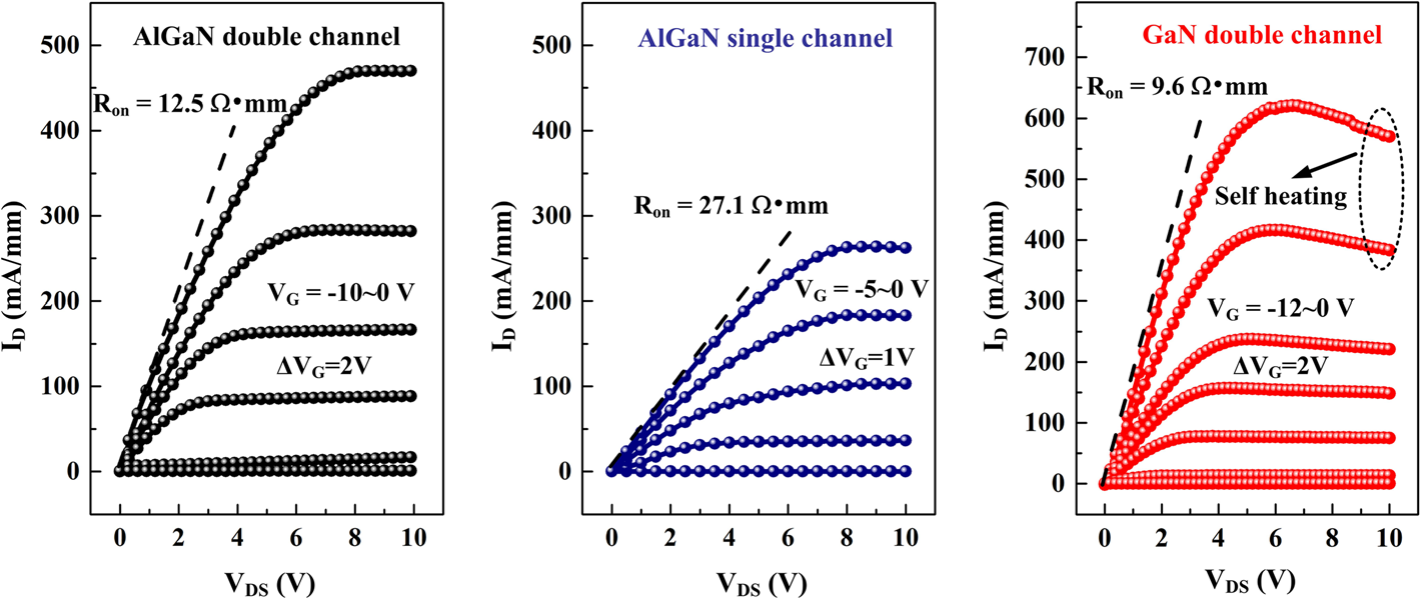
Output characteristics of AlGaN double channel, AlGaN single channel, and GaN double channel HEMTs

The typical output characteristics of the HEMTs are illustrated in Fig. 5, for which the V_GS_ and V_DS_ were swept from 0~− 10 V and 0~10 V. The maximum drain current density (*I*_max_) and differential on-resistance (*R*_on_) of the AlGaN single channel sample are 265.3 mA/mm and 27.1 Ω∙mm, respectively. These results are in accordance with the previous reports, suggesting the deficiency of AlGaN channel HEMTs in current drive capacity. For the AlGaN double channel HEMTs, the *I*_max_ dramatically increases to 473 mA/mm, which is 1.8 times higher than that of AlGaN single channel HEMTs. We attribute the improvement in *I*_max_ to the superior transport properties of the AlGaN double channel heterostructure. On one hand, double channel structure possesses two potential wells along the vertical direction, and the carrier storage capability of the AlGaN conduction channel is promoted. On the other hand, although the total channel carrier density is increased, the average electron density in each channel is reduced. As a result, the carrier-carrier scattering effect is suppressed and the channel electron mobility is elevated. However, it can be observed that the *R*_on_ of AlGaN double channel HEMTs is 12.5 Ω∙mm, still larger than that of GaN double channel HEMTs. This phenomenon is related to the large contact barrier height of the AlGaN barrier layers, for which the AlN composition is as high as 31%. Due to the self-heating effect resulted from the high power dissipation, the negative differential resistance effect can be observed for the GaN double channel HEMTs when *V*_GS_ >− 4 V and *V*_DS_> 6 V. Nevertheless, for the AlGaN channel HEMTs (both single channel and double channel), this negative differential resistance effect is significantly suppressed, manifesting the superior performance of AlGaN channel HEMTs in elevated temperature conditions.

**Fig. 6:**
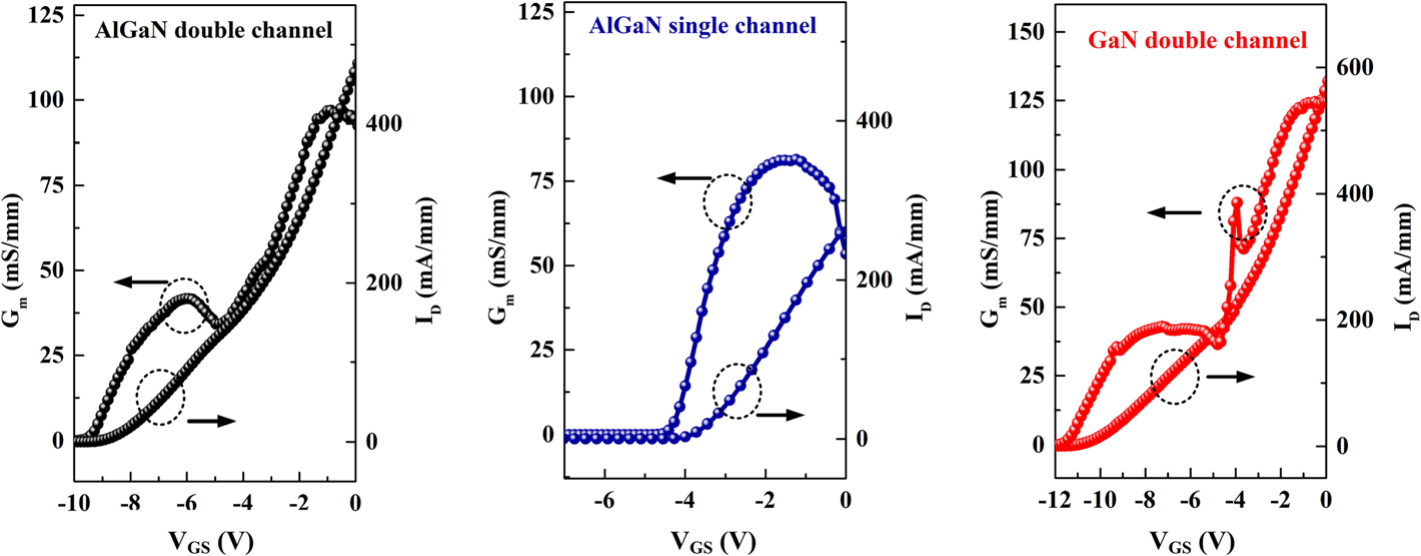
Transfer characteristics of AlGaN double channel, AlGaN single channel, and GaN double channel HEMTs

Figure 6 illustrates the typical transfer properties of the HEMTs with *V*_DS_ of 10 V. The AlGaN single channel HEMTs exhibit a threshold voltage (*V*_T_) of − 3.8 V, together with an inferior peak extrinsic transconductance (*G*_m,max_) of 80.5 mS/mm in the vicinity of *V*_GS_ = − 1.5 V. For the AlGaN double channel and GaN double channel HEMTs, the *V*_T_ remarkably decreases to − 9.2 and − 11.2 V, which is resulted from the high channel carrier density and the relatively long distance from the gate electrode to the lower 2DEG channel. The high *V*_T_ may result in high power loss of the devices at off state, and this issue can be improved by further optimizing the growth parameters of double channel structures, such as properly reducing the thickness of barrier and upper channel layers. Specially, double-hump characteristics can be observed of the transconductance curves of AlGaN double channel and GaN double channel HEMTs. For the AlGaN double channel HEMTs, two peak values of 97.9 and 42.5 mS/mm can be extracted at *V*_G_ = − 1.0 and − 6.0 V. The sub-peak value reaches 43% of the G_m,max_, indicating the decent gate-control ability and linearity of the AlGaN double channel HEMTs. Moreover, based on our previous research achievement [21], the results can be further improved by modulating the structure parameters, such as the thickness and composition of the AlGaN double channels, and the coupling effect between the double channels and the device linearity can be enhanced.

**Fig. 7:**
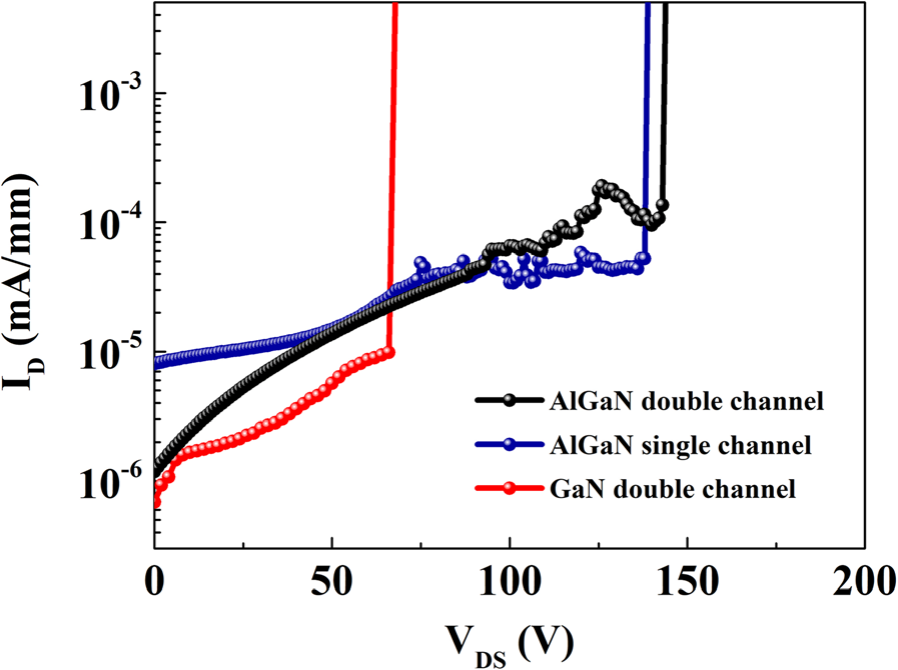
Breakdown characteristics of AlGaN double channel, AlGaN single channel, and GaN double channel HEMTs

**Fig. 8:**
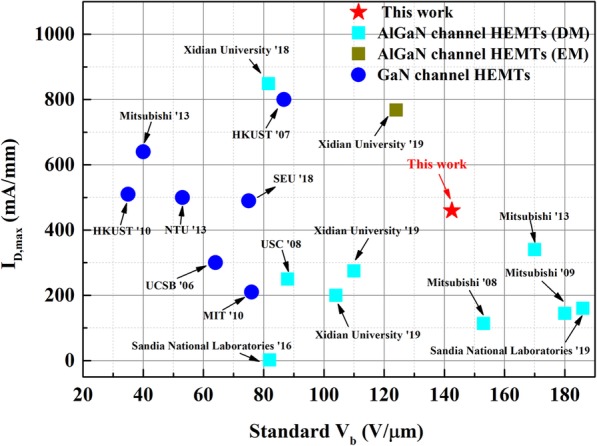
Benchmark of *I*_max_ and *V*_b,standard_ for AlGaN channel and GaN channel HEMTs

The off-state breakdown characteristics of the HEMTs based on different heterostructures are measured and shown in Fig. 7. The *V*_b_ is defined by the criteria of leakage current reaching 5 μA/mm. It can be observed that all the three samples present hard breakdown characteristics, and the breakdown performance of AlGaN channel HEMTs is obviously better than that of the GaN channel HEMTs. The *V*_b_ of the AlGaN double channel HEMTs is 143.5 V, more than two times higher than that of the GaN double channel HEMTs. Taking the *L*_GD_ = 1 μm into consideration, the *V*_b,standard_ (defined by *V*_b_/*L*_GD_) is as high as 143.5 V/μm for the AlGaN double channel HEMTs. The *I*_max_ and *V*_b,standard_ results of the AlGaN double channel HEMTs in this work are benchmarked against the GaN channel and AlGaN channel HEMTs reported by other groups in Fig. 8, and the results of depletion-mode (DM) and enhancement-mode (EM) devices are distinguished. In addition, the core indexes of the AlGaN channel HEMTs (heterostructures) in previous reports and this work are summarized in Table 1. As Fig. 8 shown, it is obvious that the breakdown performance of AlGaN channel HEMTs is generally better than that of GaN channel HEMTs, accompanying with the deterioration in *I*_max_. Noticeably, the *I*_max_ of the AlGaN double channel in this work is comparable to most results of the GaN channel HEMTs. Moreover, it is necessary to note that the *I*_max_ value in this work is obtained at *V*_GS_ = 0 V, which is conservative and can be further improved by applied positive gate voltage. Therefore, these results convincingly demonstrate the enormous potential of AlGaN double channel HEMTs in microwave power device applications.

**Table 1:** Core indexes of AlGaN channel HEMTs (heterostructures) in previous reports and this work

Institution	μ (cm^2^/Vs)	*n*_s_ (10^13^ cm^− 2^)	*I*_MAX_ (mA/mm)	*V*_T_ (V)	*V*_b,standard_ (V/μm)
Mitsubishi [6]		0.53	114		153
Mitsubishi [7]	645	0.22	145	− 1.0	180
Mitsubishi [8]	460	0.79	340	− 4.0	170
Sandia National Laboratories [9]	250	0.60	2	− 4.9	82
USC [10]	284	1.15	250	− 10	99
XDU [11]	801	0.39	200	− 4.0	104
Sandia National Laboratories [12]	390	0.72	160	− 6.0	186
XDU [13]	801	0.39	275	− 2.8	110
XDU [14]	807	0.61	849	− 4.3	82
XDU [15]	1179	0.61	768	1.0	103
This work	1130	1.30	460	− 9.2	142.5

## Conclusions

In summary, AlGaN double channel heterostructure is proposed to fabricate high performance HEMTs. The superior transport properties of AlGaN double channel heterostructure is revealed, leading to the improved current drive capability of the HEMTs. In addition, the excellent breakdown performance of the AlGaN double channel HEMTs is demonstrated. The results in this work show the great potential of AlGaN double channel devices in microwave power applications in the future.

## Data Availability

All data generated or analyzed during this study are included in this published article and its supplementary information files.

## Abbreviations

MOCVD: Metal organic chemical vapor deposition
HEMTs: High electron mobility transistors
2DEG: Two dimensional electron gas
*I*_max_: Maximum drain current density
*V*_b_: Breakdown voltage
*V*_T_: Threshold voltage
RF: Radio frequency
HRXRD: High resolution x-ray diffraction
C-V: Capacitance-voltage
*L*_G_: Gate length
*L*_GS_: Gate-source distance
*L*_GD_: Gate-drain distance
*R*_on_: On-resistance
*G*_m_: Transconductance
DM: Depletion-mode
EM: Enhancement-mode
